# Large eddy simulation of droplet transport and deposition in the human respiratory tract to evaluate inhalation risk

**DOI:** 10.1371/journal.pcbi.1010972

**Published:** 2023-03-20

**Authors:** Alicia Murga, Rahul Bale, Chung-Gang Li, Kazuhide Ito, Makoto Tsubokura

**Affiliations:** 1 Kobe University, Graduate School of System Informatics, Japan; 2 Riken Center for Computational Sciences, Japan; 3 National Cheng Kung University, Taiwan; 4 Kyushu University, Faculty of Engineering Sciences, Japan; Los Alamos National Laboratory, UNITED STATES

## Abstract

As evidenced by the worldwide pandemic, respiratory infectious diseases and their airborne transmission must be studied to safeguard public health. This study focuses on the emission and transport of speech-generated droplets, which can pose risk of infection depending on the loudness of the speech, its duration and the initial angle of exhalation. We have numerically investigated the transport of these droplets into the human respiratory tract by way of a natural breathing cycle in order to predict the infection probability of three strains of SARS-CoV-2 on a person who is listening at a one-meter distance. Numerical methods were used to set the boundary conditions of the speaking and breathing models and large eddy simulation (LES) was used for the unsteady simulation of approximately 10 breathing cycles. Four different mouth angles when speaking were contrasted to evaluate real conditions of human communication and the possibility of infection. Breathed virions were counted using two different approaches: the breathing zone of influence and direction deposition on the tissue. Our results show that infection probability drastically changes based on the mouth angle and the breathing zone of influence overpredicts the inhalation risk in all cases. We conclude that to portray real conditions, the probability of infection should be based on direct tissue deposition results to avoid overprediction and that several mouth angles must be considered in future analyses.

## Author summary

We have experienced the devastating nature of airborne transmitted diseases through the recent COVID-19 pandemic. To eradicate short and long-range cross-infection of viruses like SARS-CoV-2 and other such diseases, we need to unravel the fundamentals of droplet generation, transport and deposition due to human interactions. Here, we have computationally simulated viral droplet transport from one talking person to another and calculated the infection probability by predicting the exact number of virions attached to the respiratory tract tissue of the possible host. Our results emphasize the need of using real-like settings and conditions for virtual infection risk prediction to avoid under or overestimation. The ability to accurately predict infection probability of SARS-CoV-2 and other airborne transmitted diseases through computer simulations benefits society by enabling us to create new guidelines and preventive measures for present and future needs.

This is a *PLOS Computational Biology* Software paper.

## 1. Introduction

As evidenced during the SARS-CoV-2 worldwide pandemic that began to spread in late 2019, respiratory infectious diseases are a significant threat to human health. According to mounting evidence [1–3], one of the main routes of SARS-CoV-2 infection is airborne transmission due to the aerosols and droplets carrying viral RNA, which was later recognized by the World Health Organization (WHO) in April 2021 [4]. For the past few years, aerosols and droplets generated by expiratory events–i.e. coughing, sneezing, talking or breathing–have been primarily studied, with coughing and sneezing as the main targets because they are overt clinical signs of illness as well as eruptive sources [5–8].

In contrast, smaller droplets generated by talking have gained less attention, arguably because they are invisible to the naked eye and their impact is not as straightforward. These speech-generated aerosols and droplets, which start with a high aqueous fraction before shrinking due to evaporation, can stay airborne for longer periods while carrying SARS-CoV-2 RNA [9] and other pathogens such as influenza [10] and tuberculosis [11]. Although after evaporation these droplets are named droplet nuclei, in the present study they are referred to as “particles” for simplicity. These particles are potentially more dangerous because they can reach further into the respiratory tract for infection [12] and because a conversation or speech event usually lasts longer than a one-time cough or sneeze event, generating a larger amount. It has been proven that the amplitude of vocalization during speech, i.e. loudness, has an important part on particle emission rate and particle size distribution [13], making whispering less dangerous in terms of infection risk than a loud conversation.

Notably, the assessment of the mentioned infection risk requires the detailed study of the respiratory system, the first line of interaction between the environment and the human body. Particles generated from an infected, loudly speaking person enter the human body through inspiration and are thereafter deposited on the nasal mucosae (diameter≥10 μm), carried to the trachea and bronchi (5 μm ≤diameter≥10 μm) or inhaled directly into the lungs (diameter≤5 μm) [14].

While many studies have applied computational methods for the detailed analysis of particle transport due to expiratory activities, they have primarily focused on eruptive events [15–17] and the prediction of mean flows for engineering applications through Reynolds-Averaged Navier-Stokes (RANS) modeling [18,19] or several combinations of both [20–22].

Although these studies provide valuable information in understanding expiratory events as well as particle transport generated by them, particle trajectory and consequent deposition in the respiratory tract represent further challenges that have yet to be considered. First, by considering a fully incompressible approach, only a fundamental, unreal behavior of flow and, consequently, particles can be achieved. Second, previous research has focused on one head/mouth position for infection risk analysis. However, mouth movement/direction as a social cue is common during a conversation and must also be integrated. Third, as we have previously published [23], particle deposition is affected by the averaging of the flow done under RANS modelling and a large eddy simulation (LES) approach needs to be applied for the accurate tracking and deposition of particles.

In the present work, we have focused on two main objectives in order to expand previous research. First, we tracked particles generated from a loudly speaking human until the vicinity of a second breathing human. Here, we explain the minutiae of particle transport near the breathing zone of human beings. Second, we have analyzed the subsequent particle deposition inside the respiratory tract of the breathing human due to inhalation. In this way, inhalation risk has been calculated considering natural breathing conditions.

## 2. Methods

### 2.1. Domain design

**Fig 1:**
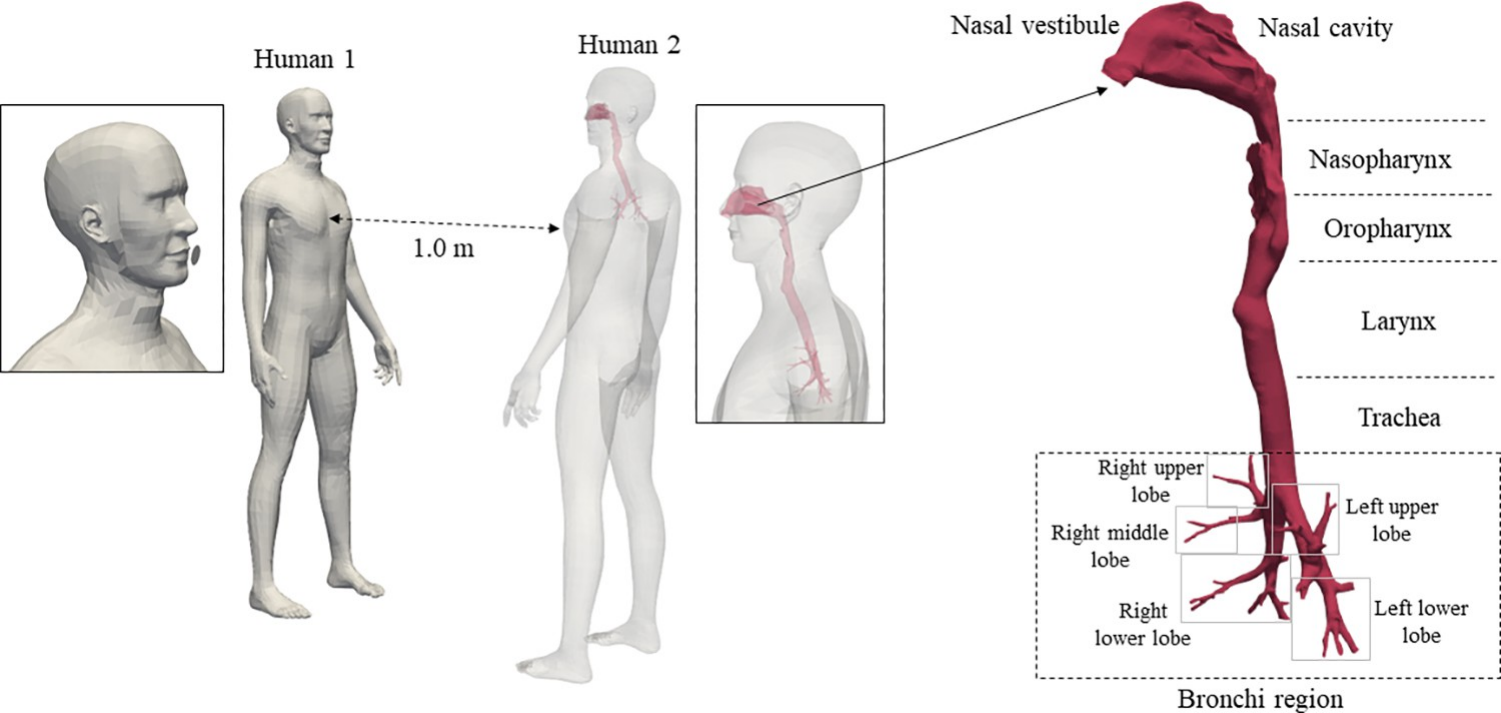
Design of analyzed domain with respiratory tract divisions explained.

The geometry used in this study consisted of two male human bodies [24], standing face-to-face and separated by a 1 m distance in an 8×8×8 m^3^ domain. The two virtual manikins complied with the guidelines proposed by Yoo and Ito [25]. “Human 1” had a simplified circular mouth geometry for a loud speaking setting, while “Human 2” was integrated with a respiratory tract for continuous breathing, provided and previously validated by Phuong and Ito [26], as presented in Fig 1. This respiratory tract was further simplified by removing the oral cavity to minimize cell number, starting from the nasal vestibule until the 7^th^ generation of bronchial tubes (4^th^ bifurcation).

**Fig 2:**
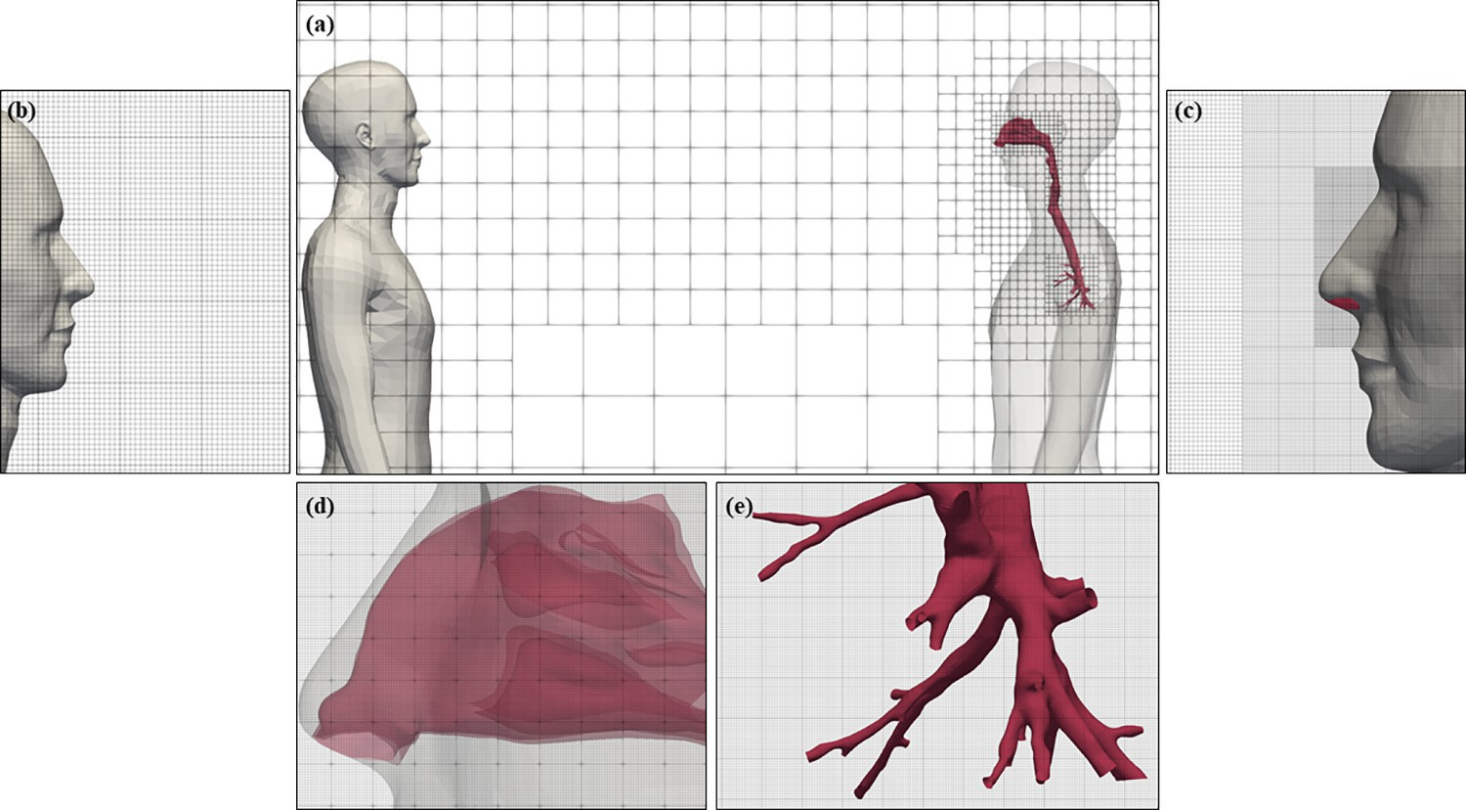
BCM mesh design (a) between Human 1 and Human 2, (b) near the mouth of Human 1, (c) around the nose of Human 2, (d) inside the nasal cavity and vestibule and (e) inside the bronchial tubes.

The building cube method (BCM) [27], which divides the domain into regions–“cubes”–and creates equidistant orthogonal cells in each cube, was used for mesh generation. This technique allows high-speed grid generation for complicated shapes, ensuring adaptability for local flow characteristics as well as high order spatial accuracy and easy parallelization because each cube contains the same number of cells. In this study, one cube was divided into 16×16×16 cells (3D), generating 103 million elements. Cell size at the nares, in the nasal vestibule, nasal cavity and nasopharynx was less than 0.48 mm to resolve the flow near the complex geometry of the cartilage and bone that comprise the nasal septum, interior canals, roof and lateral conchae (Human 2). The same size was maintained for the bronchial tubes, larynx and trachea. To resolve the loud speech jet, cell size was maintained at 3.84 mm near the mouth of Human 1 and along the jet path until reaching Human 2. Cubes mesh design is depicted in Fig 2A while cell design at different locations is shown in Fig 2B, 2C, 2D and 2E.

The human and respiratory tract geometries were modeled as immersed boundaries, where the object geometries in stereolithography format were immersed in the BCM uniform mesh and the interface cells were evaluated by using interpolation, as explained in detail by Li et al [28].

### 2.2. Boundary and numerical flow conditions

In this study, no natural or mechanical ventilation settings were considered, assuming the ambient wind as static, where the flow velocity was V_flow_ = 0.0 m/s. Human body surface temperature was set constant at 300 K while respiratory tract tissue temperature was set at 309 K, roughly core body temperature. Ambient temperature (*T*) was 297 K with a relative humidity of 60%. In this case, the flow was considered primarily convective. Regarding wall surface treatment, all the immersed boundaries were set with the no-slip condition and the outer boundaries of the domain were treated with the slip condition. Turbulence was solved using an implicit LES method, further explained in section 2.3. Initial pressure was set to 101.3 kPa. This study uses a sufficiently small time-step, to satisfy the Courant-Friedrichs-Lewy (CFL) condition, set to 0.001 seconds.

#### 2.2.1. Speaking model

**Fig 3:**
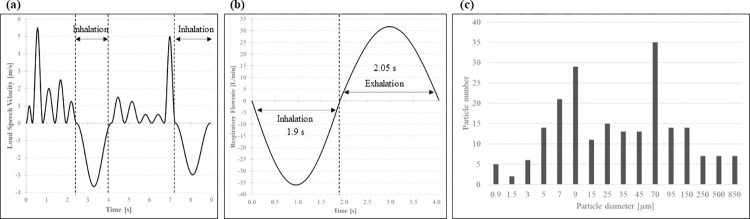
(a) Speaking model over one cycle, (b) breathing model over one cycle and (c) particle diameter distribution used for ejection.

The loud speech velocity profile (S1 Data) from Human 1 is shown in Fig 3A. This profile was generated when counting from 1 to 10 in English and conformed to a sinusoidal model, based on the experimental study by Gupta et al [29]. Period 1 (counting from 1 to 5) was between 0.0 s and 2.4 s while period 2 (counting from 6 to 10) was between 4.1 s and 7.2 s. Transitional inhalation periods to maintain mass balance of the exhaled air were introduced after period 1 and period 2. In this study, one speech cycle consisted of period 1, period 2 and the two transitional inhalation periods, completing a total of 9 seconds. The flow velocity was amplified by 50% for loud speech and generated from the simplified, circular mouth geometry (diameter = 6 cm^2^) which was located roughly 1 cm from the mouth-wall of Human 1. Initial temperature and relative humidity of the exhaled air were set at 305 K and 95%, respectively. This condition was set at the simplified mouth surface of Human 1 as a transient velocity profile, with a 0.01 interval, based on the above experimental results.

#### 2.2.2. Breathing model

For the respiratory condition in Human 2, the breathing profile was also based on Gupta et al. [29]. Fig 3B shows one respiratory cycle, consisting of an inhalation period between 0.0 and 1.9 s and an exhalation period between 1.9 and 4.05 s. in terms of volume flow rate in liters/minute [L/min] (see S2 Data). The flow was defined by a sum of sine equations proposed by Gupta et al [29]. and further corrected by Kadota el al [30]. The sum of equations and metabolic parameters used to construct the breathing cycle used in this study are listed below.


$$Q=\alpha_{x}sin(\beta_{x}t)$$ (1)

$$\alpha_{x}=\frac{\beta_{x}TV}{2}$$ (2)

$$\beta_{x}=\frac{\pi {RF}_{x}}{30}$$ (3)

$${RF}_{in}=55.55-32.86H+0.2602W$$ (4)

$${RF}_{out}=77.03-45.42H+0.2373W$$ (5)

MV=(0.0826M+1.271)×BSA (6)

$$TV=\frac{MV({RF}_{in}+{RF}_{out})}{2{RF}_{in}{RF}_{out}}$$ (7)

Where the breathing flow rate *Q* [L/min] is determined by a sinusoidal wave and *α*_*x*_ and *β*_*x*_*t* embody the amplitude and period of the wave during inhalation or exhalation (_*x*), respectively. *TV* is the tidal volume [L/breathing cycle], RF is the respiration frequency [times/min] for inhalation or exhalation (_*x*) and MV is the volume per minute [L/min]. MV and RF vary according to subject, organ of breathing or human posture, the so-called metabolic parameters represented by H, body height [m]; W, body weight [kg]; M, metabolic rate [W/m^2^] and BSA, body surface area [m^2^]. In this study the metabolic parameters have been set to 1.736 m, 66.3 kg (body mass index = 22 kg/m^2^), 60 W/m^2^ for a standing person at rest and 1.94 m^2^_,_ respectively.

The velocity profile set at the nostrils’ surface was straightforward, calculated based on the above equations and the nares’ surface area of 2.4 cm^2^, with an interval of 0.01 seconds. A dynamic anatomic pulmonary model was applied to the bronchial tubes to divide the bronchial tree into independent lung segments for heterogeneous ventilation, based on the study by Shelley et al [31]. In this manner, the respiratory flow entering from the trachea into the multiple bronchi was transformed into fractional alveolar flow at each lung segment (i.e. lobe) and uniformly distributed using lobe height, proportional to the ventilation rate. The sum of equations used to divide the flow and calculate the partition rate at each lobe are listed below and the lung segment division has been explained in Fig 1.


$$\dot{V}=-0.00031h+0.064$$ (8)

$$R_{p}=\frac{\sum \dot{V}}{N}$$ (9)

$$Q_{b}=Q\times R_{p}$$ (10)

Where $\dot{V}$ is the fractional alveolar flow at each lung lobe; *h* is the height of the lung lobe from the lungs, already calculated by Shelley et al(31).; *R*_*p*_ is the partition ratio of each lobe; *N* is the total number of bronchi in the lobe; *Q* is the breathing flow rate calculated in Eq (1) and *Q*_*b*_ is the final fractional airflow at each bronchus. Both *Q* and *Q*_*b*_ were carefully calculated to maintain mass balance during the breathing cycle and bronchus velocity was calculated based on the transversal area of each end. The discharged air during the exhalation period was considered adiabatic. A velocity profile for each bronchus, corresponding to the fractional airflow calculated by Eq (10) and transversal area of each tube was set every 0.01 seconds at the bronchus’ surface.

#### 2.2.3. Particle model and particle size distribution

During vocalization, the acoustic waves of pressurized air pass through narrow folds at high-speed. As these folds part, the rushing air breaks the fluid filaments and films formed between the parting surfaces of tongue, lips and teeth, fragmenting them into droplets that join the exiting airstream [32]. The size of the droplets varies according to the place of generation: particles originated from the oral cavity tend to be bigger while the size of those generated at the vocal folds oscillates between 1–5 μm [33,34]. In this study, the initial droplet diameter distribution follows the model proposed by Duguid [35] and has been modified for loud speaking by increasing droplet count by a factor of 1.5 for droplets with a diameter less than 20 μm and a factor of 7 for diameters larger than 70 μm with a linear increase for intermediate values, as previously explained by Bale et al [36]. Fig 3C shows the initial distribution of droplet diameter (S3 Data).

A simple sputum droplet model is adopted in this work wherein the sputum droplet is assumed to be composed of water and virions. After evaporation, the nonvolatile components, namely viral particles, remain and are called droplet nuclei. For simplification, droplet-nuclei are called “particles” in this study.

We have carefully followed quality control practices and checked the numerical/boundary conditions. Furthermore, the validation of the evaporation model has been done by comparing the evaporation of single droplet to the experimental data of Ranz and Marshall [37,38] and has been previously reported by Bale et al [36].

#### 2.2.4. Mouth angle

**Fig 4:**
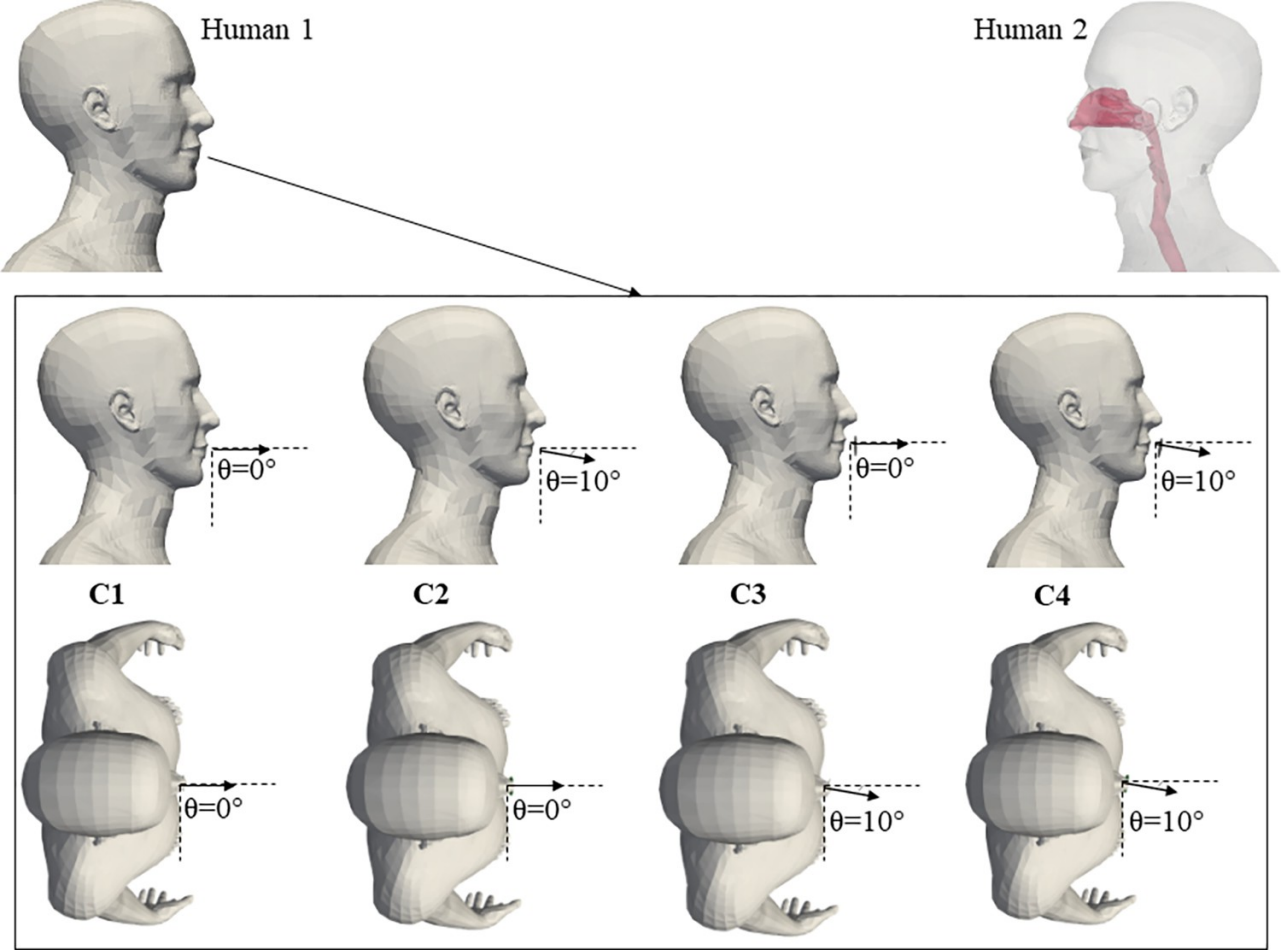
Angle variations of mouth for cases C1 to C4.

Although numerous studies have mixed the mouth position in a fully frontal direction for loud speech, head and mouth movements are not stationary, as explained by Tiede et al [39]. These types of movements can structure the discourse, indicate deixis and multiple lexical connotations. Moreover, mouth/head movement, angling and position are closely related to motoric speech production: adjustment for respiration, amplitude and peak loudness [40–42]. In this study, we have considered four instances of mouth angle variation for Human 1, which represent mouth and head movement during loud speech. A minimal angle of 10° has been considered to quantify how even slight changes in position affect inhalation risk. Cases 1 to 4 (C1–C4) are illustrated in Fig 4.

### 2.3. Governing equations

The flow solver used in the present study is named CUBE [28] (Complex Unified Building cubE), based on the finite volume method and primarily designed for high performance computing application. This framework solves the compressible fluid flow using an Eularian approach and the particle dynamics using a Lagrangian frame while the immersed boundary bodies are discretized into material/Lagrangian particles attached to its surface. An implicit method has been applied to solve the sub-grid turbulence scale, where the implicit dissipation provided by the convective numerical scheme is equivalent to the dissipation provided by conventional, explicit sub-grid scale models [43,44].

The governing flow Eqs in compact vector notation are:

$$\frac{\partial U}{\partial t}+\nabla ∙F=S$$ (11)

Where **U**, represents the primitive flow variables, **F** is the combined convective and diffusive flow and **S** is the source term. **U** and **F** are further explained as:

$$U=(\begin{array}{c} \rho \\ \rho u_{1}\\ \rho u_{2}\\ \rho u_{3}\\ \rho e\\ \rho Y_{k} \end{array}),\;F_{i}=(\begin{array}{c} \rho u_{i}\\ \rho u_{i}u_{1}+P\delta_{i1}-\mu A_{i1}\\ \rho u_{i}u_{2}+P\delta_{i2}-\mu A_{i2}\\ \rho u_{i}u_{3}+P\delta_{i3}-\mu A_{i3}\\ \rho (\rho e+P)u_{i}-\mu A_{ij}u_{j}+q_{i}\\ \rho u_{i}Y_{k}-\rho {\hat{u}}_{i}^{k}Y_{k} \end{array})$$ (12)

Where *ρ* is the gas density, *μ* is the viscosity and *u*_*i*_, *e*, and *P* are the velocity, total specific energy and pressure, respectively. The subscripts 1, 2 and 3 represent the components of the velocity along the principle directions. *Y*_*k*_ and ${\hat{u}}_{i}^{k}$ are the species mass fraction, **q** = −*λ*∇*T* is the heat flux vector where *λ* and *T* are the diffusivity and temperature, respectively, and *A*_*ij*_ represents the stress tensor. The density and pressure are given by the state equation *P* = *ρRT*, where *R* is the gas constant and *T* the temperature. *e* is given by:

$$e=\frac{P}{\gamma -1}+\frac{1}{2}u_{i}u_{i}$$ (13)

Where *γ* is the heat capacity ratio.

Finally, the diffusion velocity ${\hat{u}}^{k}Y_{k}$ is given by the relationship ${\hat{u}}^{k}Y_{k}=D_{k}\nabla Y_{k}$, where *D*_*k*_ is the species diffusivity. The source term **S** for species transport corresponds to the evaporation of the droplet nuclei, referred to as “particles” in this study for simplification, and given by the following vector:

$$S=(\begin{array}{c} 0\\ (\rho -\rho_{0})g_{1}\\ (\rho -\rho_{0})g_{2}\\ (\rho -\rho_{0})g_{3}\\ (\rho -\rho_{0})g_{i}u_{i}\\ S_{\rho }Y_{k} \end{array})$$ (14)

Where *ρ* and *ρ*_0_ are the local and far field ambient density and **g** is the gravitational acceleration.

#### 2.3.1. Particle transport and evaporation

In this study, a one-way particle-flow analysis was considered where the carrier flow was not impacted by the mass and momentum of the particles; therefore, feedback to the flow due to particle motion was neglected. Particles were treated through a discrete Lagrangian approach while the flow was considered Eularian. Sputum particle dynamics were analyzed through the single droplet model, where other forces were neglected due to the small size of the particles and particle density (1000 kg/m^3^) [45]. This study uses LES to directly predict turbulence fluctuations, overcoming the necessity of using indirect stochastic methods for particles and independent turbulent eddies interactions [46]. The present single droplet model was calculated through the following Eqs:

$$\frac{{dx}_{d}}{dt}=u_{d}$$ (15)

$$\frac{{du}_{d}}{dt}=\frac{{3C}_{D}}{{4d}_{d}}\frac{\rho }{\rho_{d}}(u-u_{d})|u-u_{d}|+g$$ (16)

Where **x**_*d*_ and **u**_*d*_ are the position and velocity of a single particle, respectively; *d*_*d*_ is the particle diameter; *ρ*_*d*_ represents its density and *C*_*D*_ is the drag coefficient, expressed as a function of the particle Reynolds number and given by $Re=\rho max(|u-u_{d}|)d_{d}/\mu$. For *Re*<1000, *C*_*D*_ was given by $C_{D}=\frac{24}{Re}(1+\frac{1}{6}{Re}^{2/3})$; and for *Re*>1000 it was set at 0.424.

The change in particle mass due to evaporation is expressed as:

$$\frac{{dT}_{d}}{dt}=\frac{Nu}{3Pr}\frac{c_{p}}{c_{l}}\frac{f_{1}}{\tau_{d}}(T-T_{d})+\frac{1}{m_{d}}(\frac{{dm}_{d}}{dt})\frac{L_{v}}{c_{p,d}}$$ (17)

$$\frac{{dm}_{d}}{dt}=-\frac{m_{d}}{\tau_{d}}(\frac{Sh}{3Sc})\ln\left(1+B_{M}\right)|D_{d}>D_{particle}$$ (18)

And *D*_*d*_≤*D*_*particle*_ for aerosolization

Where *T* is the ambient temperature, *T*_*d*_ is the particle temperature, calculated through convective heat transfer with ambient air and the evaporative heat loss; *B*_*M*_ is the mass transfer number *B*_*M*_ =(*Y*_*v*,*s*_−*Y*_*v*_)/(1−*Y*_*v*,*s*_), defined by the local mass fraction of the particle at the surface *Y*_*v*,*s*_ and the far field *Y*_*v*_ as well as the velocity; *Sh* is the Sherwood number, given by the particle Reynolds number *Re* and by the Schmidt number *Sc*, $Sh=2+0.552{Re}_{d}^{1/2}{Sc}^{1/3}$; *m*_*d*_ is the particle mass and *L*_*v*_ is the evaporation latent heat. *c*_*p*_ is the specific heat at constant pressure and *c*_*l*_ is the specific heat capacity of the particle. *τ*_*d*_ is the particle response time. Under the simple sputum droplet model, we assume that the droplet is composed of water and virions. After the evaporation of the volatile components, the droplets turn into aerosol particles composed of only virions. As typical coronaviruses sizes are in the range of 80 to 120 nm, we chose an aerosol particle size of 1 μm, allowing for up to several hundred virions in an aerosol particle. Evaporation in Eq 18 is carried out until particle diameter is reduced to *D*_*particle*_ = 1*μm*. Thereafter, only the particle transport equation is solved for the aerosolized particle. Finally, particles were evenly injected from the simplified mouth surface of Human 1 into the domain at the times of peak velocities consistent with the speech model presented in Fig 3A, with a velocity and direction corresponding to those of the flow.

Further mathematical details and terms used for this model have been reported by Bale et al [47,48].

## 3. Results and discussion

In this study, simulations were performed for 49.0 s and particles were injected from 9.0 s to allow flow field development due to loud speech. Particle travelling time from Human 1 to Human 2 was roughly 3.0 s and particle inhalation started from 12.0 s, which was the first inhalation period after particle release. Thus, the flow inside the respiratory tract was developed for three breathing cycles before initial particle inhalation to avoid hysteresis as suggested by Inthavong et al [49].

### 3.1. Loud speaking, mouth angle and generated flows

**Fig 5:**
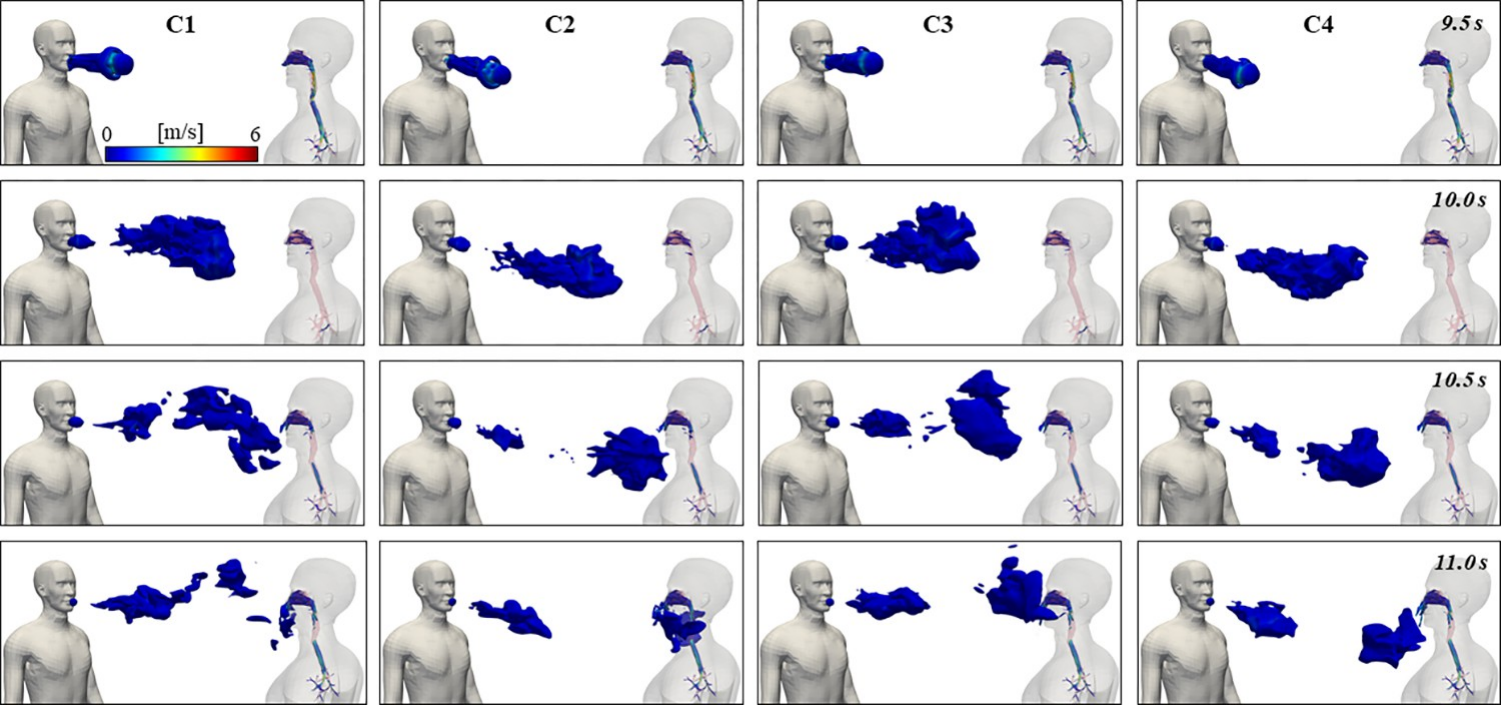
3D depiction of speech jet generated by Human 1 over one cycle and inhaled/exhaled air by Human 2 for all cases.

Fig 5 evidences the changes in exhaled flow from 9.5 s to 11.0 s, which correspond to the speech jet created during the third speaking cycle of this study. These results show that the jet originating from the mouth, similarly to all jets generated through an orifice, produces vortices which drive the transport around the head. The exhaled flow rate varied with time in accordance to sound productions and intonation from the model presented in Fig 3A. The four different mouth angles generated different exhale directions for the starting jet at 9.5 s, although the starting penetration distance was maintained. The jet is followed by trains of puffs from 10.0 s, which are a product of the interrupted jet due to the consequent rapid releases of air during speaking, previously described by Bourouiba et al. [50] and Abkarian et al. [51]. The LES approach applied here resolved the transient, large turbulent eddies that influence the transport of particles contained inside each puff, which reached the breathing zone of Human 2 differently according to the mouth angle. The puff reached the face of Human 2 in C1, when the inclination angle was 0° in all directions but passed to the left of Human 2 face when the inclination angle was 10° in all directions, contrasting the influence of mouth angle and puff trajectory. For all cases the jet was turbulent, which allows for easier mixing of particles with the surrounding environment and consequent transport and the continual speech cycles increase these features. Velocity distribution outside of the wake flow region formed by the speech-breathing interaction had no significance due to the stagnant nature of the proposed flow (V_flow_ = 0.0 m/s). Thus, most particles were entrapped in the wake region, not following this secondary flow. A strong recirculation zone downstream Human 2 was generated, especially for C3 and C4. The convective predominance of the flow can be seen in the wake region between Human 1 and Human 2, where horizontal flow recirculation is directed to the centerline between these two, conveying extra momentum and accelerating this flow, along with particle transport.

### 3.2. Air inhalation through breathing

**Fig 6:**
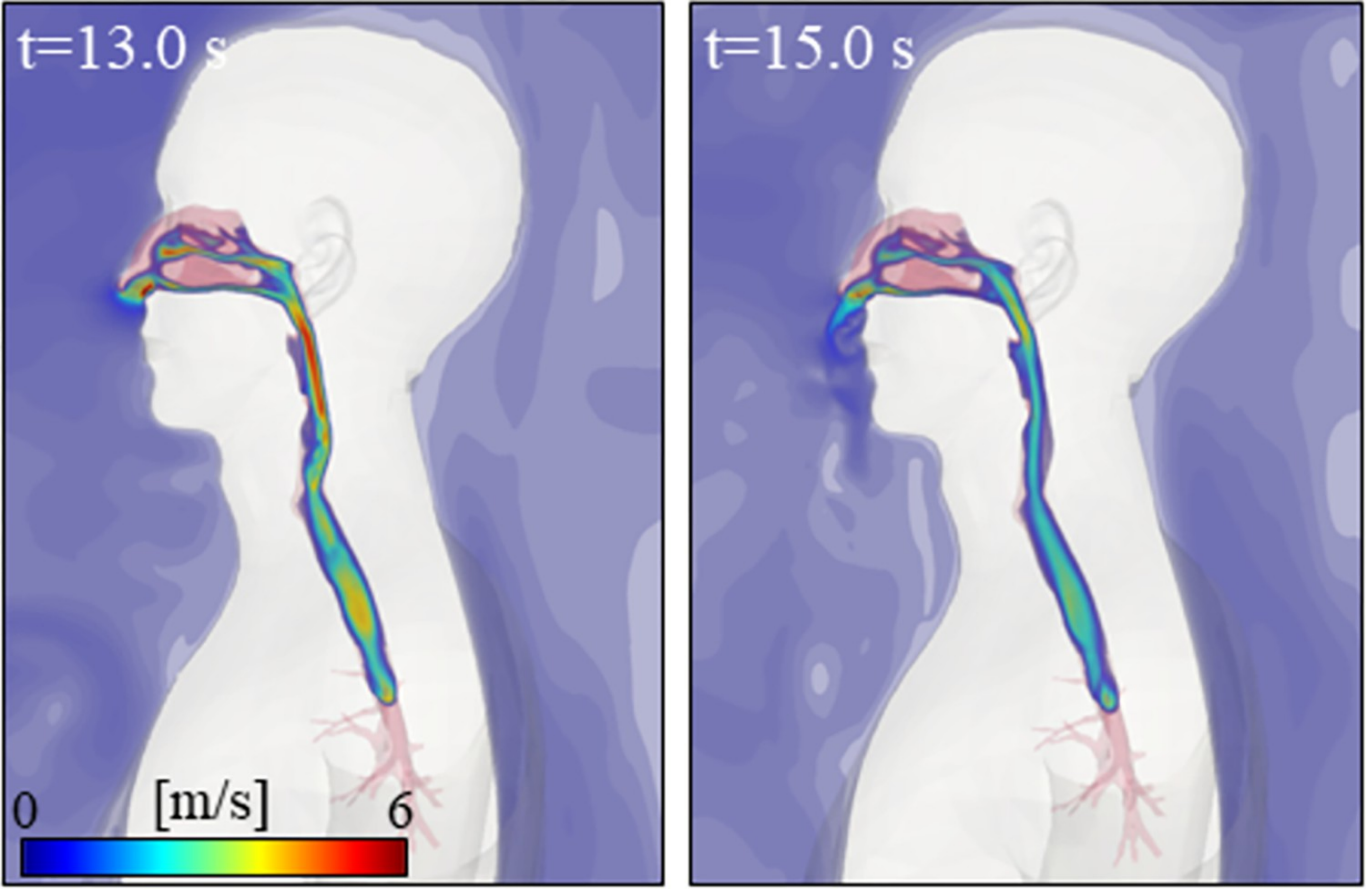
Instantaneous distributions of inhaled and exhaled air by Human 2 at t = 13.0 s and t = 15.0 s for C1.

Fig 6 shows distributions of air inside the respiratory tract for case C1 to C4 at 13 seconds and 15 seconds, which roughly correspond to the highest inhalation and exhalation points of the 4^th^ breathing cycle, respectively. No differences in flow pattern inside the respiratory tract (inhalation or exhalation) were found between C1 and C2, C3, C4. In this situation, the flow generated by human speech has negligible impact on the biological airflow generated by autonomous human breathing. In contrast, the development of the exhaled air jet, outside of the human body and closest to the nasal breathing area, was directly influenced by the speech flow generated by Human 1. High velocity magnitude was found inside the nasal vestibule, nasal cavity and nasopharynx due to the complex geometry. During inhalation (t = 13.0 s), nasal jets hit the superior conchae, driving high-speed flows in the nasal septum. The separated flow merges at the Y-shaped bifurcation of the nasopharynx, gaining intensity as it hits the posterior wall of the oropharynx and finally forming vortices as it travels along the trachea. Although maximum velocity was generated at the oropharynx constriction, moderate velocity was formed at the lower region of the trachea. During exhalation (t = 15.0 s), flows at the mid-trachea are similar to the previously described ones, confirming the auto-regulation of human body functions where the glottal contraction is able to control flow characteristics induced by the outdoor environment.

### 3.3. Exhaled particles during loud speech

**Fig 7:**
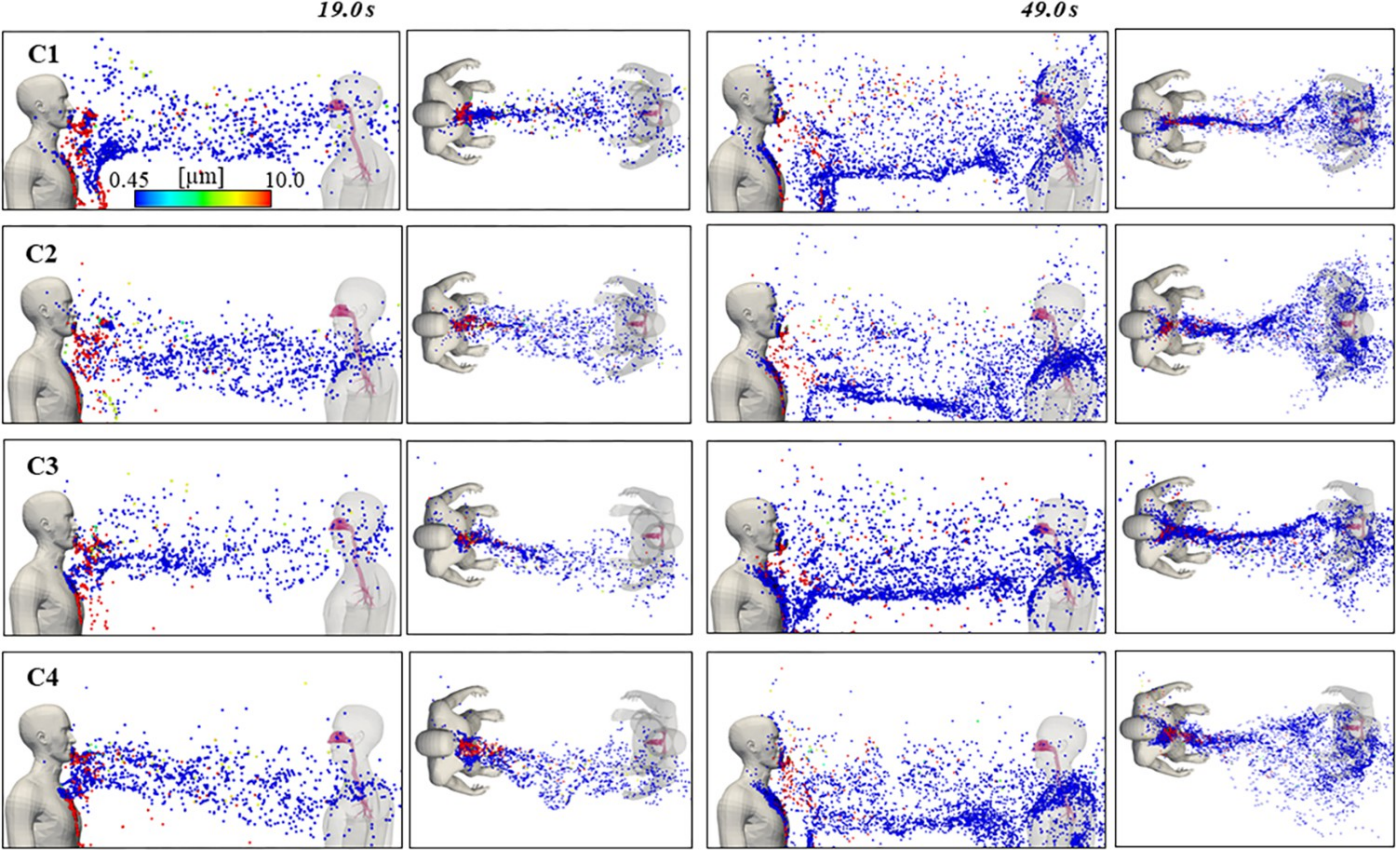
Instantaneous states of particle dispersion at t = 19.0 s and t = 49.0 s for all cases with particles colored according to their diameter.

In this study, initial particle injection started from 9.0 s and continued at each velocity peak of exhalation, according to the model shown in Fig 3A. Vertical and horizontal instantaneous states of particle dispersion are presented in Fig 7 at 19.0 s and 49.0 s, after 10 seconds and 40 seconds of initial particle injection, respectively. Roughly all particles with diameter larger than 20μm fall to the ground rapidly within the range of the starting jet due to the effect of gravity. Smaller particles remain airborne and are transported within the consequent train of puffs. Due to the absence of a ventilation current and air motion due to other speakers, the exhaled particles largely reach the face of Human 2 in C1, with a modest angle to the left in the breathing zone of Human 2, likely due to the influence of its inhalation/exhalation. Particles show a marked downward trend when the vertical inclination was 10° (C2 and C4), remarking the influence of starting jet direction, train of puffs and particle trajectory. The 10° horizontal inclination of the mouth in C3 and C4 affected considerably the initial trajectory of the particles, seen at 19.0 s and consequently decreased the number of particles near the face of Human 2 at the final state of the calculation. Cloud puff direction was largely dependent on mouth angle and particle accumulation due to the continuous speech was present. Finally, puff trend direction of C1 was compared to literature sources–numerically and experimentally–under similar conditions for particles mostly under 1 μm, where the puff train also presented a slight left-side deviation of Human 1 [15,52].

### 3.4. Quantifying the number of particles in the breathing zone

**Fig 8:**
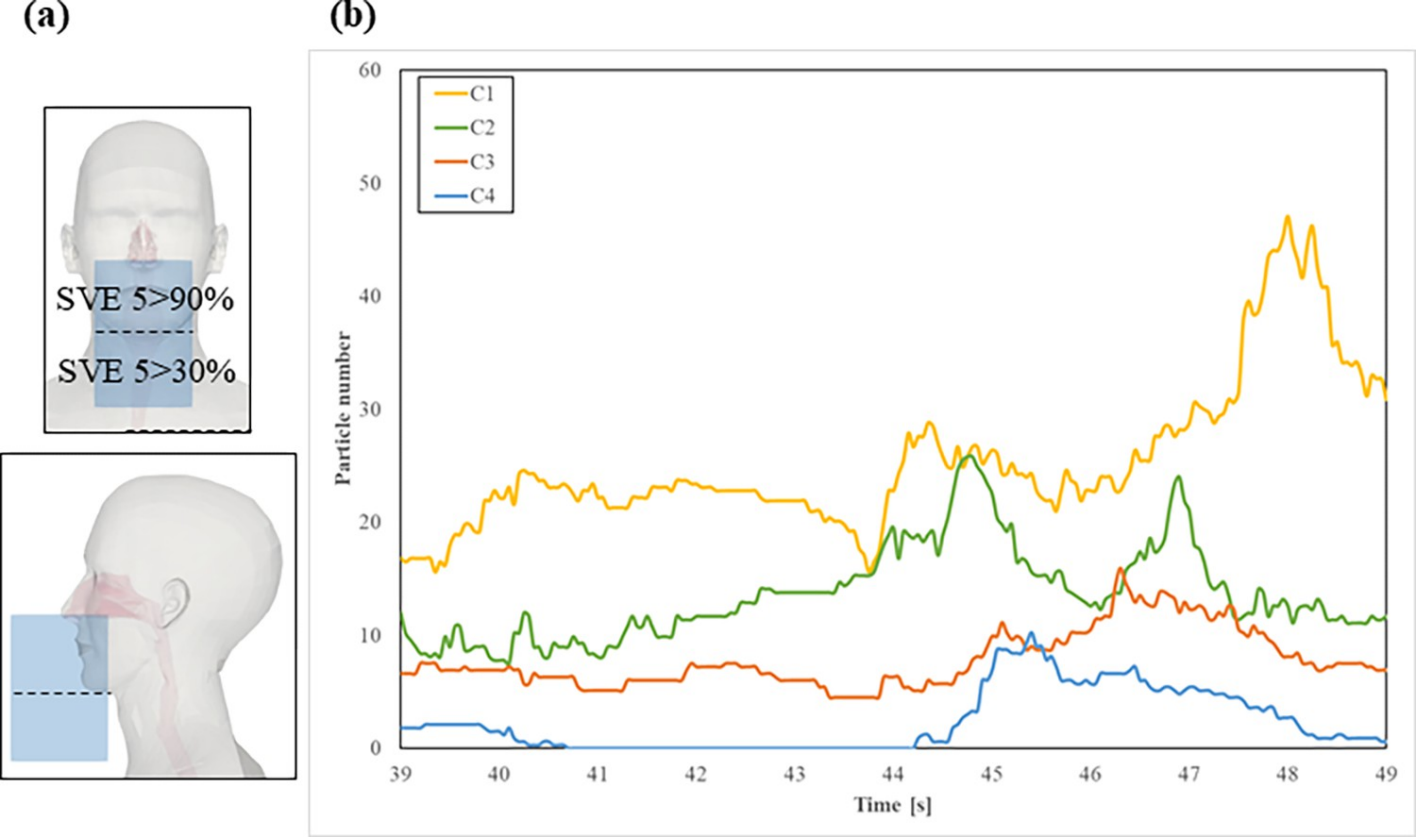
(a) Depiction of breathing zone box according to SVE 5 and (b) number of particles in the breathing zone from t = 39.0 s to t = 49.0 s.

The breathing zone is commonly described as the area around the nose and mouth where most of the air is inhaled, although no uniform definition has been reached. To evaluate infection risk and quantify particle residence, this study defines the breathing zone based on the transient portion of air vented and exhausted from the nose, according to the model proposed by Kuga et al [53]. This model predicted the probability of inhaled air distribution from the nose to the chin as > 90% and below the chin as > 30%, according to the scale for ventilation efficiency 5 (SVE 5) proposed by Kato [54,55]. Two rectangles with dimensions 10×10×7 cm^2^ and 10×10×8 cm^2^ for SVE 5 > 90% and SVE 5 > 30%, respectively, were created according to Fig 8A. Boxes dimensions were chosen according to the approximate order of inhalation/exhalation of Human 2 with the longer side being placed from nose to ground according to common breathing direction and were centered around the nose. Particle residence corresponding to the sum of both rectangles for the last 10 seconds of calculation was monitored in order to compute time-averaged values, as shown in Fig 8B (S4 Data). Particle residence was higher for C1 because the puffs containing the particles directly reached the face of Human 1 while C4 had the lowest residence due to the 10° tilt of the mouth (horizontal and vertical direction). A 10° downwards mouth tilt (C2) showed a decrease in particle residence due to the influence of gravity. For all cases, the turbulent nature of the flow shows a varying number of particles residing in the breathing zone resulting from transverse spreading and mixing of the exhaled puffs. Particle residence accumulate in the environment over time due to the continual exhalation cycles of Human 1. Similar particle behavior in the breathing zone for C1 was found in other literature: only airborne particles with a diameter less than 2 μm were found near the nose at the last stage of simulation time when static ambient air was considered [56].

To further quantify the number of particles in the breathing zone, we contrast cases C1 to C4 by calculating particle rate [particles/s] and mass rate [μg/s] in the breathing zone (Table 1). C1 had higher particle rate, and therefore mass rate, while results for C4 were almost negligible with a minimum tilt of 10° angles in all directions. Mass rate for C2 and C3 was roughly comparable, suggesting that a mouth tilt in only one direction yields similar particle residence results.

**Table 1:** Particle and mass rate in the breathing zone and deposited in the respiratory tract.

	Particle rate [particle/s]		Mass rate [μg/s]	
*Case*	*Breathing zone*	*Respiratory tract*	*Breathing zone*	*Respiratory tract*
C1	2.6	1.3	0.04	0.02
C2	1.4	0.4	0.02	0.01
C3	0.8	0.5	0.02	0.01
C4	0.2	0.1	0.00	0.00

### 3.5. Direct respiratory tract deposition

**Fig 9:**
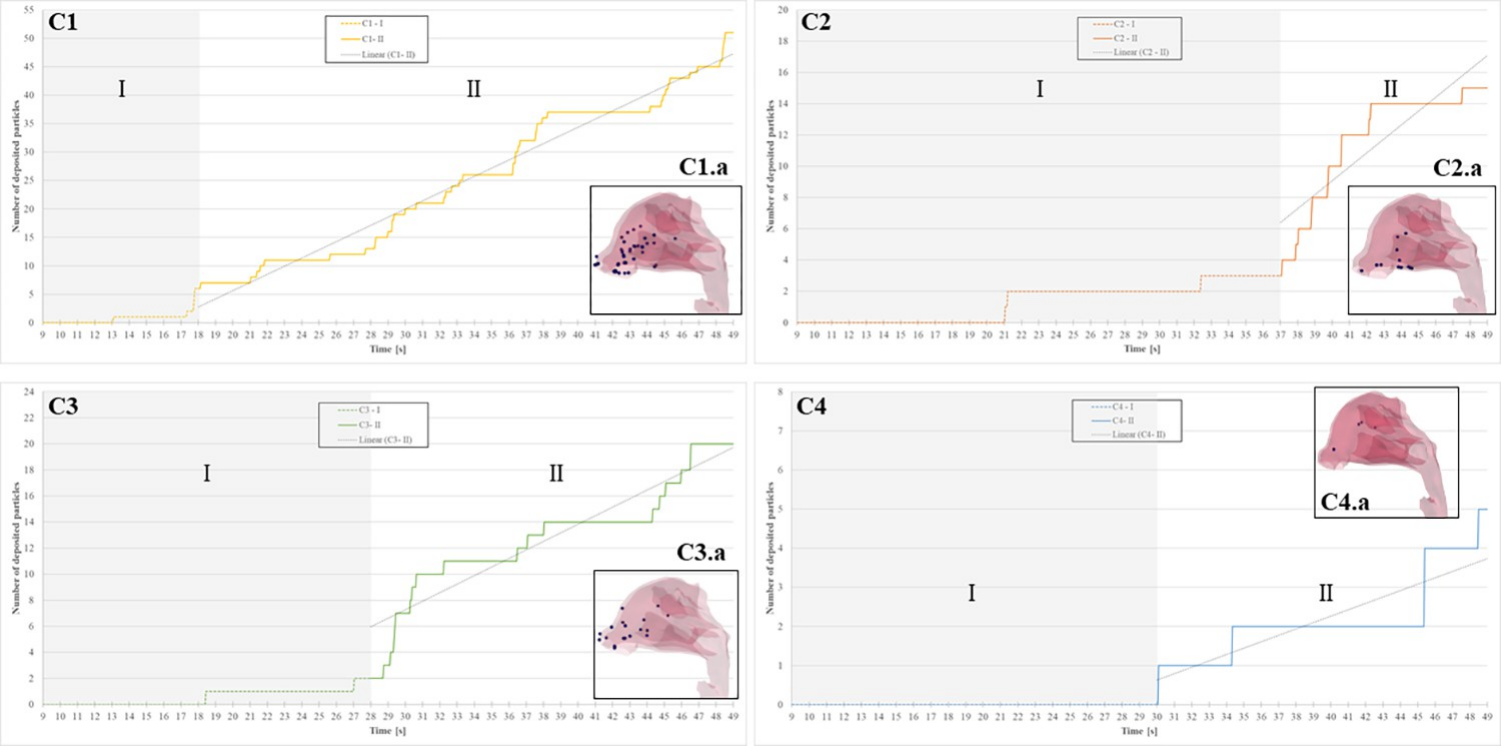
Evolution of particle deposition in the nasal vestibule and nasal cavity and depiction of total number of particles deposited in the respiratory tract.

Fig 9 shows results of particle deposition on the upper region of the respiratory tract cut roughly at the end of the nasopharynx for all cases, along with a depiction of the final state of particle deposition at 49.0 seconds on the right of each graph. Here, the detailed behavior of particle deposition can be studied. Once the process of evaporation occurred, all particles reported the same uniform diameter = 0.9 μm due to mathematical constraints in the CUBE framework. At this range of diameter, inertial impaction and interception are dominant features of particle deposition and were therefore deposited in the vicinity of the nasal vestibule and nasal cavity in all cases. Similar trends were found in literature when compared to the present study, where droplets deposited mainly in the nasal cavity due to dominant inertial impaction [33,57–59].

The graphs present the fundamental trends of temporal evolution of particle deposition in the respiratory tract for all cases (see S5 Data). This evolution shows two main phases: Phase I represents the “spreading phase”, from particle injection through travelling time until reaching Human 2 and the subsequent slow rate of particle deposition. Phase II represents the state where enough particles are present in the air and the gradient of particle deposition is more or less constant, where a linear fit has also been profiled. While C1 presents a rapid increase of particle deposition, the number of inhaled particles was lower in all other cases, especially in C4. Again, temporal evolution for cases C2 and C3 were similar, confirming that the mouth tilt in one direction produces equivalent results of particle deposition. The difference in mouth angle generates mass rate variation due to the first angle of penetration of the particle through the nares, which, along with particle characteristics, will affect directly the primary features of deposition in the human body: impaction, interception, sedimentation and diffusion.

We have also quantified the particle rate and mass rate of deposition, also presented in Table 1 to contrast both calculation approaches. We found that the predicted mass rate in the breathing zone was double the amount of the mass rate deposited in the respiratory tract for all cases, which directly affects the evaluation of the inhalation risk.

### 3.6. Evaluation of infection risk

Finally, although more exhaustive models of infection risk have been proposed [60], we have calculated the inhalation risk based on the infection probability dose-response model proposed by Wells [61] and improved by Riley [62] due to its simplicity, defined as:

$$P=1-e(-\frac{N_{x}}{N_{0}}I)$$ (19)

Where *P* is the probability of infection, expressed in percentage [%]; *N*_0_ is the number of virions needed for infection [copies], assumed as 900 copies in this study, which lies within range of previously reported values [36,63]; and *I* is the infectivity of the viral strain. *I* = 1 (100%) corresponds to the original strain of SARS-CoV-2 while the infectivity of delta and omicron strains are higher by 145% (2.45) and 267% (3.67), respectively. *N*_*x*_ is the number of inhaled virions based on the number of inhaled particles according to breathing zone residence (*N*_*in*_) or the number of deposited particles in the respiratory tract (*N*_*dep*_) which we have calculated as:

$$N_{in}=\frac{(\frac{\rho_{vD}}{\rho })V_{in}P_{in}}{T_{in}}\gamma t$$ (20)

$$N_{dep}=\frac{\gamma }{\rho }nt$$ (21)

Where *γ* is the viral load (1×10^13^ copies/m^3^); *ρ* is the density of the saliva particles (1000 kg/m^3^); *n* is the particle deposition rate (Table 1); and *t* is the exposure time assumed as 15 minutes in this study. *ρ*_*vD*_ is the droplet density in the breathing zone, calculated as the time-averaged particle mass for the last 10 seconds divided by the volume of the assumed breathing box presented in Fig 8; *V*_*in*_ is the volume of inhaled air, *P*_*in*_ is the proportion of inhalation in a breathing cycle and *T*_*in*_ is the inhalation period in one cycle.

Table 2 shows the results of infection probability for the three SARS-CoV-2 strains calculated from the two approaches above: 1) by quantifying the number of particles in the breathing zone, and b) by directly calculating the number of deposited particles in the respiratory tract of Human 2. No vaccination effect has been considered in this study.

**Table 2:** Infection risk SARS-CoV-2 according to breathing zone residence and respiratory tract deposition.

Case	Infection probability					
	Original		Delta		Omicron	
	*Breathing zone*	*Respiratory tract*	*Breathing zone*	*Respiratory tract*	*Breathing zone*	*Respiratory tract*
**C1**	30%	17%	58%	37%	73%	50%
**C2**	18%	13%	39%	28%	53%	39%
**C3**	16%	10%	36%	23%	48%	33%
**C4**	2%	1%	4%	4%	7%	6%

The infection probability of cases C1 to C4 changes drastically with a minimum mouth tilt of 10° in the specified directions. While assuming a 0° tilt case (C1) shows basic information, the inclusion of realistic mouth angle variation allows for the exact prediction of inhalation risk, which must be as precise as possible to formulate adequate prevention procedures and guidance. Calculating the infection probability based on the number of particles in the breathing zone overpredicts the risk for all cases. One of the reasons for this overestimation is that quantifying particle residence in the breathing zone adds the particles deposited on the alae (sidewalls of the nostrils), which in reality do not increase infection risk.

These results offer significant understanding on the minutiae that needs to be considered when calculating inhalation risk and infection probability as well as demonstrate the importance of mouth angle variation and exact prediction of particle deposition in the human body versus particle quantification in an assumed “breathing box zone”.

## 4. Validation

**Fig 10:**
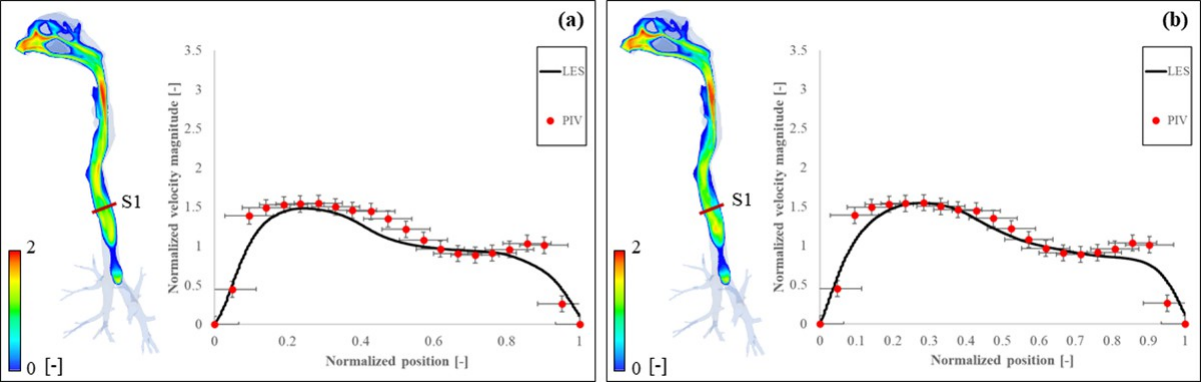
Airflow distribution and PIV comparison for (a) cell size≈0.40 mm and (b) cell size = 0.20 mm in the respiratory tract.

In order to validate the calculation of internal airflow in the respiratory tract, a separate numerical simulation containing only the respiratory tract at constant inhalation (15 L/min) was performed and compared to experimental PIV data previously published by Phuong and Ito [26], as shown in Fig 10 (S6 Data). The normalized velocity profile (*U*_*n*_ = *U*/*U*_*in*_) at the cross-sectional location S1 was plotted against the position, made non-dimensional by dividing it by the diameter of the trachea at that location (*x*/*D*). As grid independence check, 2 simulations under the same conditions were carried out: Fig 10A shows the airflow distribution and PIV comparison when the cell size was roughly the same as the one used in the present study (≈0.40 mm) and the trachea was discretized by approximately 50 elements while Fig 10B shows airflow results and PIV comparison for cell size = 0.20 mm and roughly 100 elements for trachea discretization. In both cases, distribution results were consistent with those previously published [26,64]. Regarding the comparison with PIV data, results presented slightly larger discrepancies for normalized positions between 0.9 and 1.0 due to flow separation, especially in Fig 10A, where grid size was bigger. This flow separation occurred near regions with complicated geometry containing curves and bends, affecting the calculation of the boundary layer. In this instance, a finer mesh can capture the near-wall flow more accurately, as depicted in Fig 10B. Although we have considered both cases as reasonably consistent with PIV measurements, further mesh refinement is required to eliminate flow detachment.

## 5. Applications and limitations

### 5.1. Applications

The present observations offer insights into the risk of airborne transmission, demonstrating the importance of two main, but not widely considered, factors: 1) biologically-precise interactions between the infected person and possible host, and 2) head movement while speaking. The methodology developed in this study can be used to establish bounds for doses in corresponding intervals of sought responses for airborne diseases like COVID-19, tuberculosis or influenza. By also considering the role of the surrounding environment, the presented framework can be used in advance indoor air quality assessment and design targeting the mitigation of airborne infections instead of just minimizing energy consumption, a necessary evolution in the post-pandemic society. Furthermore, by confirming the relationship between head angle and infection risk, this study offers fundamental information on weighted-averaging to reflect multiple head angles when predicting infection risk for realistic head movement during loud speech.

### 5.2. Limitations

There are several limitations to this study. First, all simulations were performed for 40 seconds after injection (49 seconds total) but further simulation time is needed for meaningful statistical results. Second, the distance between Human 1 and Human 2 was set to 1 meter but a parametric study should be performed in the future to enrich the knowledge of social distance corroboration. Third, minimum head movement of 10° was analyzed to confirm its impact on infection risk. However, more instances of head rotation should be considered due to the fact that, when speaking, duration of fixed head position occurs only for short periods. For this same reason, a time-weighted average of head movement should also be considered. Finally, although validation for particle transport and airflow inside the respiratory tract was performed, no validation of particle deposition in the respiratory tract was possible because of the lack of experimental and literature data available.

## 6. Conclusion

This study predicted infection probability of a breathing person caused by an infected, loudly speaking person situated at a 1-meter distance from another. This exact value of infection risk was calculated by carefully transporting the exhaled particles contained in the speech puffs until they reached the breathing zone of the target individual. These particles have been quantified in the carefully defined breathing zone and thereafter transported inside the human body by an inhalation/exhalation model based on metabolic parameters. The number of particles and their individual mass deposited in the nasal vestibule and nasal cavity have been measured and then compared to the number of particles residing in the breathing zone. Based on these two contrasting approaches, we have also predicted the probability of infection and we found that the probability of infection is always overestimated when considering the breathing zone of influence. On the other hand, the probability of infection calculated using the results of direction deposition can lead to more realistic suggestions. Furthermore, this study has also demonstrated the influence of the mouth tilt during speech and the subsequent infection probability. Considering only a completely vertical position for the mouth, as is the case in many studies, leads to inhalation risk uncertainties, which directly become a health issue due to proposals of imprecise guidelines for asymptomatic patients.

The purpose of this study was to contrast the prediction of inhalation risk based on the generalized assumption of particle residence in the breathing zone against detailed results of particle deposition in the respiratory tract while at the same time analyzing the effects of mouth angle while speaking. Although the small number of cases and inhaled particles limit the statistical contribution, the results we have presented show the relevance of using realistic metabolic parameters, particle transport models, physiological behavior models and actual considerations of human interaction to predict infection probability.

## Supporting information

S1 Data Loud speaking model. Velocity [m/s] profile used as boundary condition in Human 1 from 0.0 to 100.0 seconds, spaced every 0.01 seconds.
(XLSX)

S2 Data Breathing flowrate model. Flow rate [L/min] used for respiration boundary condition in Human 2, from 0.0 to 100.0 seconds, spaced every 0.01 seconds, along with the metabolic parameters used to calculate it based on the model proposed by Gupta et al. (29).
(XLSX)

S3 Data Particle injection model. Initial distribution of particle diameter [mm] presented in Fig 3, as well as particle injection time from 9.0 to 100.0 seconds.
(XLSX)

S4 Data Particle vs time (BZ). Number of particles counted in the breathing zone from 39.0 to 49.0 seconds, as explained in section 3.4.
(XLSX)

S5 Data Particle vs time (Dep.). Particles deposited inside the respiratory tract.
(XLSX)

S6 Data Validation. Comparison of airflow profile at a determined location between simulations results for course and finer mesh against experimental PIV results by Phuong and Ito (26)
(XLSX)
